# Supplementary figures and images for: Estimation of supply and demand for public health nurses in Japan: A stock-flow approach
Source: PLoS One. 2025 Feb 3;20(2):e0313110. doi: 10.1371/journal.pone.0313110 (PMC11790149; doi:10.1371/journal.pone.0313110)

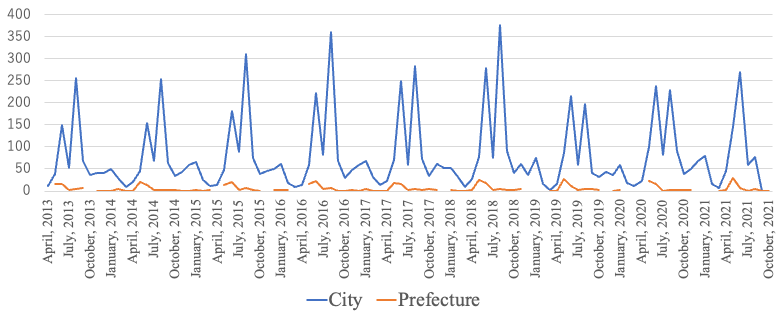


**S1 Fig. Trends in the demand for employment of public health nurses by month from 2013 to 2021.**

Supplement: S1 Fig — (DOCX) [file pone.0313110.s001.docx]

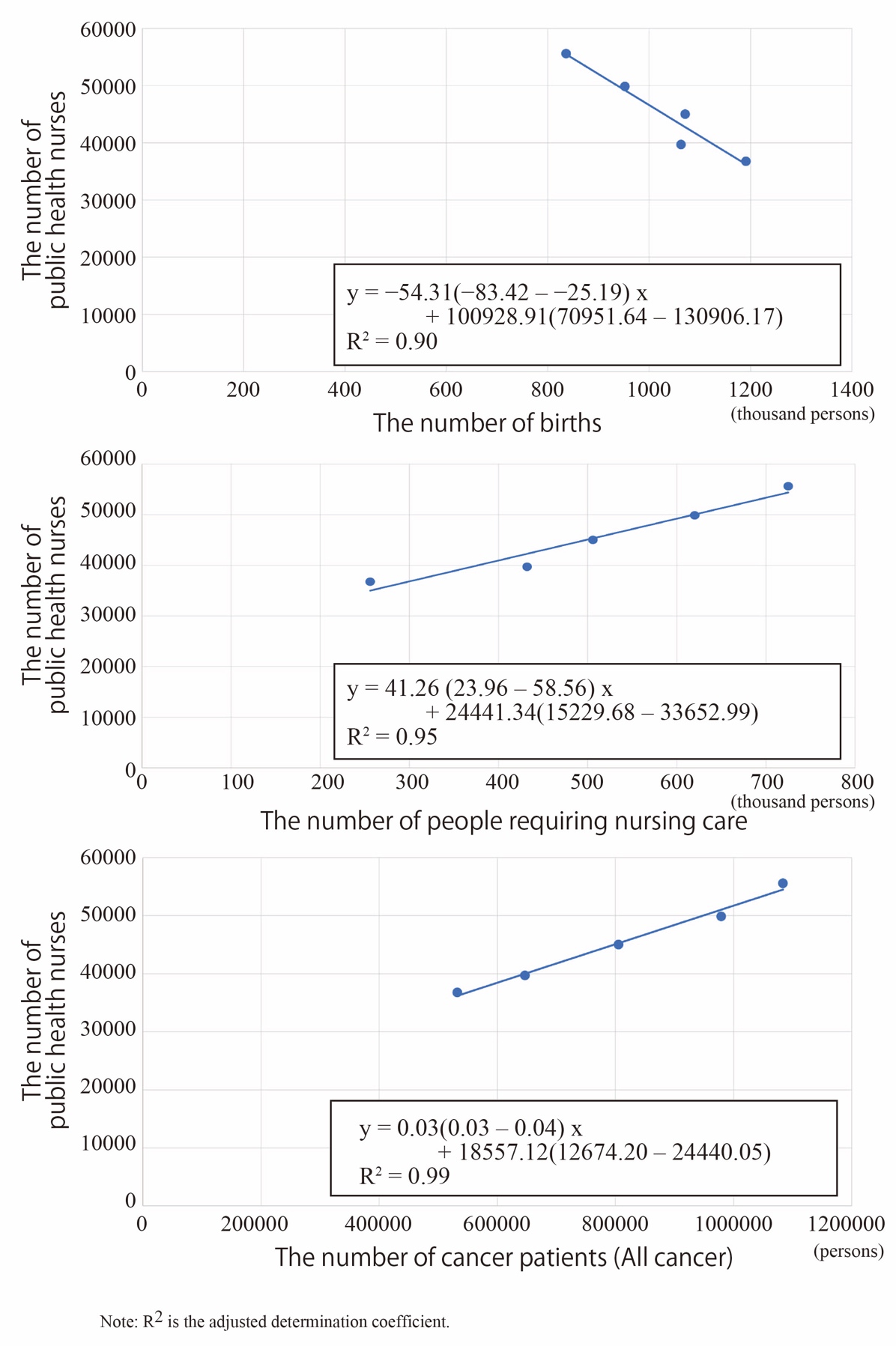


**S3 Fig. Correlations between demographic variables and the number of public health nurses**

Supplement: S3 Fig — (DOCX) [file pone.0313110.s003.docx]
