# Supplementary material for: Estimation of supply and demand for public health nurses in Japan: A stock-flow approach
Source: PLoS One. 2025 Feb 3;20(2):e0313110. doi: 10.1371/journal.pone.0313110 (PMC11790149; doi:10.1371/journal.pone.0313110)
Supplement: S2 Fig — (DOCX) [file pone.0313110.s002.docx]

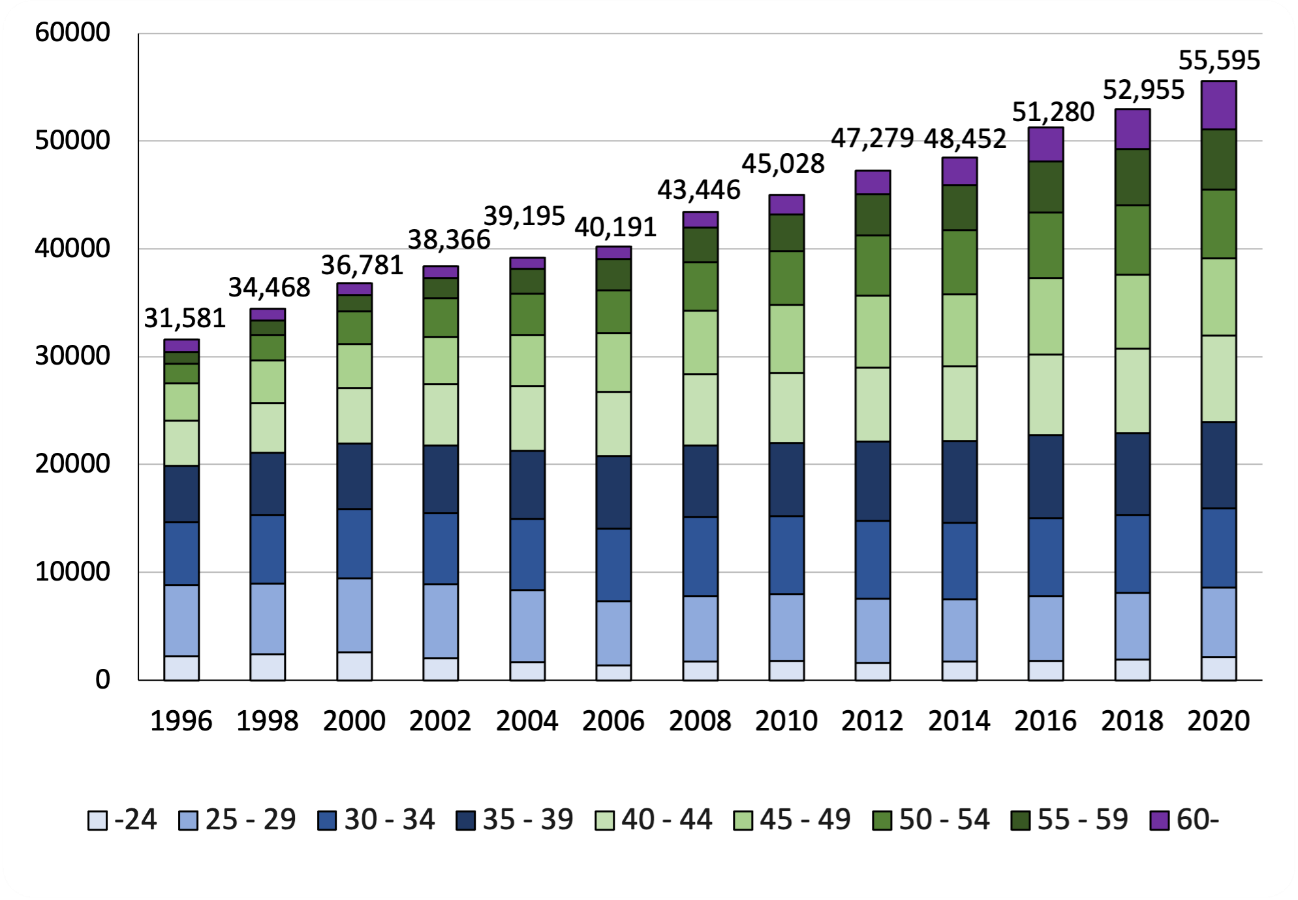


**S2 Fig. Changes in the distribution of public health nurses working in local governments by age group**
