# Supplementary material for: Estimation of supply and demand for public health nurses in Japan: A stock-flow approach
Source: PLoS One. 2025 Feb 3;20(2):e0313110. doi: 10.1371/journal.pone.0313110 (PMC11790149; doi:10.1371/journal.pone.0313110)
Supplement: S4 Fig — (DOCX) [file pone.0313110.s004.docx]

**Survey on the Employment Status of Public Health Nurses (PHNs)
 during the Corona Disaster Survey Questionnaire**

**Name of your municipality ( )**

**Name of department in charge ( )**

**<** **Recruitment information for full-time PHNs and registered nurses (RNs) >**

1. **Please tell us the number of full-time PHNs and RNs employed and working in your municipality in each of the last five years. Please fill in the number of PHNs and RNs as of April 1 for each year.**

|  | Fiscal Year (FY) 2017 | FY 2018 | FY 2019 | FY 2020 | FY 2021 |
| --- | --- | --- | --- | --- | --- |
| Number of full-time employed PHNs (as of April 1) |  |  |  |  |  |
| Of which, number of PHNs taking maternity or paternity leave |  |  |  |  |  |
| Of which, number of PHNs taking sick leave |  |  |  |  |  |
| Number of full-time employed RNs (as of April 1) |  |  |  |  |  |
| Of which, number of RNs taking maternity/paternity leave |  |  |  |  |  |
| Of which, number of RNs taking  sick leave |  |  |  |  |  |

Note: Please record maternity, childcare, and sick leave regardless of the length of time taken, as long as the leave is actually taken.

1. **Please tell us the number of recruitment examinations and the number of full-time PHNs hired in the past five years. If multiple recruit examinations are conducted, please enter the total number of recruits for all examinations, and if the number of recruits for FY 2021 is not yet determined, please provide the expected number of recruits.**

|  | FY 2017 | FY 2018 | FY 2019 | FY 2020 | FY 2021 |
| --- | --- | --- | --- | --- | --- |
| Number of recruitment examinations conducted |  |  |  |  |  |
| Number of hired PHNs |  |  |  |  |  |
| Of which, number of new graduates hired^※^ |  |  |  |  |  |

　　　*New graduate hires are defined as hires who have graduated from a public health nurses training school or graduate school and have

no previous work experience in other municipalities, hospitals, or companies.

1. **Please tell us about the number of full-time PHNs who had left the workforce in the past five years. For FY2021, please provide the number of PHNs leaving as of the end of September.**

|  | FY 2017 | FY 2018 | FY2019 | FY 2020 | FY 2021 |
| --- | --- | --- | --- | --- | --- |
| Number of full-time PHNs who had left the workforce |  |  |  |  |  |
| Of which, number of mandatory retirements |  |  |  |  |  |
| Number of full-time RNs who had left the workforce |  |  |  |  |  |
| Of which, number of mandatory retirements |  |  |  |  |  |

**< Recruitment information for part-time PHNs and RNs>**

1. **Please tell us the number of part-time PHNs and RNs hired in each of the last five years.**

**If the number of PHNs or RNs hired in FY2021 is not yet determined, please indicate the number of them expected to be hired.**

|  | FY 2017 | FY 2018 | FY2019 | FY 2020 | FY 2021 |
| --- | --- | --- | --- | --- | --- |
| Number of part-time PHNs hired |  |  |  |  |  |
| Number of part-time RNs hired |  |  |  |  |  |
