# Supplementary material for: Estimation of supply and demand for public health nurses in Japan: A stock-flow approach
Source: PLoS One. 2025 Feb 3;20(2):e0313110. doi: 10.1371/journal.pone.0313110 (PMC11790149; doi:10.1371/journal.pone.0313110)
Supplement: Attachment — Submitted filename: Response_to_Reviewers.docx [file pone.0313110.s005.docx]

Dear Editors and Reviewers,

Thank you for allowing us the opportunity to review our manuscript. Also, we are glad to have constructive and educational comments from you. We believe that our manuscript has been considerably improved thanks to your comments.

Below are our point-by-point responses to your comments and the manuscript with track change is uploaded as a supplementary file. Also, we found several other improvements in the wording in addition to the points suggested in the peer review, which we have also corrected. The corrections are summarized at the end of the document. Your comments have made the paper much better and more readable for the readers. We would appreciate it if you could check the revisions.

**Response to Editor**

**1. Please ensure that your manuscript meets PLOS ONE's style requirements, including those for file naming. The PLOS ONE style templates can be found at
https://journals.plos.org/plosone/s/file?id=wjVg/PLOSOne_formatting_sample_main_body.pdf
and
https://journals.plos.org/plosone/s/file?id=ba62/PLOSOne_formatting_sample_title_authors_affiliations.pdf.**
**Response:** Yes. We checked that this paper follows the submission guidelines of PLOS ONE.

**2. Thank you for stating in your Funding Statement:
“This work was supported by FY 2021　Kusunoki 125 of Kyoto University 125th Anniversary Fund. The funders had no role in study design, data collection and analysis, decision to publish, or preparation of the manuscript.”**

**Please provide an amended statement that declares *all* the funding or sources of support (whether external or internal to your organization) received during this study, as detailed online in our guide for authors at http://journals.plos.org/plosone/s/submit-now. Please also include the statement “There was no additional external funding received for this study.” in your updated Funding Statement.**

**Please include your amended Funding Statement within your cover letter. We will change the online submission form on your behalf.**
**Response:** We appreciate the information you have provided. In the cover letter, you declared that "There was no additional external funding received for this study."

**3. Your ethics statement should only appear in the Methods section of your manuscript. If your ethics statement is written in any section besides the Methods, please delete it from any other section.**
**Response:** Thank you for pointing this out. We removed the following Ethical approval section.

**Removed: Page 23, Line 347 – 351**

**Ethical approval and consent to participate**

The ethical review of this study was waived by the Kyoto University Graduate School and Faculty of Medicine Kyoto University Hospital Ethics Committee because it was a questionnaire survey that did not involve personal information and secondary use of published data.

**Response to Reviewers**

**Response for Reviewer #1: 
#1_1. In line 215, are these the confidence intervals? Or what is the ±?**
**Response:** Thank you for your question. Following convention, the description was written in the sense of mean ± standard deviation, but to avoid misunderstanding, we have modified these sentences to clearly expression.

| **Original: Page 14, Line 214 – 218** The number of full-time PHNs was 103.2 ± 75.9 in 2019 and 104.5 ± 76.4 in 2020. The number of part-time PHNs was 11.7 ± 14.9 in 2019 and 22.4 ± 53.6 in 2020. The number of PHNs who quit or retired was 5.0 ± 4.3 in 2019 and 4.9 ± 4.5 in 2020. The number of PHN recruitment examinations was 1.3 ± 1.0 in 2019 and 1.6 ± 1.1 in 2020 (Table 1). | **Revised: Page 15, Line 230 – 236** The mean number of full-time PHNs for local governments was 103.2 (Standard Deviation: SD=75.9) in 2019 and 104.5 (SD=76.4) in 2020. The mean number of part-time PHNs for local government was 11.7 (SD=14.9) in 2019 and 22.4 (SD=53.6) in 2020. The mean number of PHNs who quit or retired for local government was 5.0 (SD=4.3) in 2019 and 4.9 (SD=4.5) in 2020. The number of PHN recruitment examinations for local government was 1.3 (SD=1.0) in 2019 and 1.6 (SD=1.1) in 2020 (Table 1). |
| --- | --- |

**#1_2. The text in table 1 is unclear**

**Response:** We apologize for the broken layout of Talbe1 in the PDF proofing. After modifying the layout, the description of the mean and standard deviation has been modified to make it easier to understand as in comment #1_1. (Table 1)

**#1_3. Could the demand estimates not be made based on all of the independent variables (birth, cancer rate etc.?) instead of providing separate estimates for each?
Response:** Thank you for your insightful comments.　We also attempted to make predictions by multivariate analysis, but the small number of data used, and the confidence intervals were very large, and the analysis deemed inappropriate (Appendix). We believe that comparing models with Adjusted R^2^ values is also beneficial because it confirms that there are no major differences between univariate and multivariate analyses, and that looking at univariates does not change the trend when forecasts are made with different variables. However, since the construction of a more accurate forecasting model is an issue for the future, we have added it to the limitations section.

Appendix. The estimated number of public health nurses by multivariate analysis

| Year | The number of  public health nurses | 95% Confidence Interval | |
| --- | --- | --- | --- |
|  |  | Lower | Upper |
| 2025 | 56227.8 | -84201.7 | 226785.8 |
| 2030 | 56054.1 | -87553.8 | 237146.9 |
| 2035 | 55982.7 | -88812.2 | 243597.5 |

**Insert: Page 24, Line 347 – 349**
Fourthly, although simple univariate prediction models were used to estimate demand of PHNs in this study, more accurate model building might be done using multivariate analysis. However, more data needs to be accumulated, and this is an issue to be addressed in the future.

**#1_4. The response rate to the survey could be discussed in the methods section.
Response:** Thank you for your advice. We have left the response rate in the Results section because we believe it is an important part of the results in determining the adequacy of the underlying data used in the estimation. If there is a reason why they should be placed in the Methods section, we would appreciate it if you could point it out again.

**#1_5. line 215: you say "the number of PHN is 103", this seems rather low, could there be a clearer way to describe what you mean?
Response:** I appreciate your honest question. We have corrected the clarifying sentences because the number you pointed out is the mean number of PHNs working in the local government where the response was received. Based on the data in Figure 2 and S2, the number of PHNs in Japan in 2020 is 55,595. If the 150 local governments surveyed in this study employed an average of 103 public health nurses each, the total number of PHN would be 15,450. This figure is considered reasonable since there are approximately 1,500 small municipalities that do not have public health centers, which were excluded from the survey. The descriptions for inclusion and exclusion criteria of local governments added based on the reviewer comment #2_3.

**Insert: Page 7, Line 110–Page8, Line 118**
Local governments where public health centers are located consist of prefectures that unite cities, towns, and villages, and cities with large populations, such as ordinance-designated cities with populations of 500,000 or more and core cities with populations of 200,000 or more. Including other smaller cities, towns and villages, there are 1,718 local governments in Japan. However, only local government with public health centers were included in this survey because large local governments, which have more discretion over personnel costs, are more sensitive to increasing or decreasing the number of PHNs, and public health centers are also likely to be significantly affected by COVID-19 pandemic.

**Insert: Page 8, Line 122–124**
However, since the objective of this study was to determine the number of staff engaged in public health nursing services, we requested that PHNs and RNs working in hospitals and clinics operated by local governments be excluded from the count.

**Insert: Page 25, Line 364–368**
Thirdly, the survey for local governments conducted in this study was conducted for prefectures and large cities and did not take into account the number of PHNs in smaller local governments. However, smaller local governments were excluded from the study because some of them have no choice for increase or decrease in the number of PHNs, with only 1 or 2 PHNs assigned to them in the first place, and they may be affected by other factors, such as the shortage of human resources in rural areas.

**#1_6 line 26 and 27, talking in terms of change rate supply and demand is confusing for me. I would expect to hear something about the gap between total demand and supply.**

**Response:** I strongly agree with your suggestion. Fig. 3 has been partially revised to show the calculation process, and Fig. 4 has been added to show the future demand and supply gap. Accordingly, the results of Fig. 4 have been added to the abstract and main text.

| **Original: Abstract** At the current job change rate, the supply of PHNs is expected to be 2,285 in 2025, 1,879 in 2030, and 1,908 in 2035. The demand for PHNs is estimated to be 2,335–2,779 in 2025, 2,314–2,886 in 2030, and 2,238–2,680 in 2035. | **Revised: Abstract** At the current job change rate to PHNs, the gaps between future supply and demand of PHNs were expected to be -494 – -50 in 2025, -1,007 – -435 in 2030, and -772 – -330 in 2035. If the job change rate would be 1.5 times, the gaps were estimated to be 285 – 729 in 2025, -31 – 141 in 2030, and -182 – 260 in 2035. If the job change rate would be 2.0 times, the supply was sufficient for all estimated years. |
| --- | --- |
| **Insert: Page 21, Line 312–Page22, Line317** Calculating the gap between supply and demand for future PHNs, the supply was insufficient for demand in all estimated years 2025, 2030, and 2035 when the current rate of job change to PHNs was used. If the job change rate to PHNs would be increased by 1.5 times, the supply was sufficient in 2025, but in 2030 and 2035, there was a shortage of up to 431 positions. Furthermore, if the job change rate would be increased to 2 times, the supply was sufficient for all estimated years (Fig 4).  Fig 4. Gaps of future supply and demand of public health nurses. | |

**Response for Reviewer #2: 
#2_1. Line 41, check and correct the mistakes in punctuation
Response:** We apologize for the typographical error, which we have corrected.

| Original: Page 3, Line 41 hospitals, most work in public health centers owned by the local government [2]. --PHNs in | Revised: Page 3, Line 43 hospitals, most work in public health centers owned by the local government [2]. PHNs in |
| --- | --- |

**#2_2. Line 48 the statement is not clear. Consider paraphrasing it
Response:** Thank you for your point in improving readability. We have added the information because we believe it is necessary to explain the qualification system for public health nurses in Japan.

| Original: Page 3, Line 47–Page 4, Line 49 Due to the nature of their duties and qualifications, PHNs also have difficulty employing foreigners like nurses and caregivers [6]. | Revised: Page 4, Line 49–53 PHNs have difficulty hiring foreigners such as nurses and caregivers because other countries do not have PHN positions that perform the same duties as in Japan or the appropriate national qualifications, and they lack a system to help foreigners obtain PHN qualification [6]. |
| --- | --- |

**#2_3. Provide more details on the selection criteria of the survey sites in the methodology section.
Response:** Thank you very much for your critical suggestion. We added the sentences regarding the selection criteria for target local government, PHNs and RNs to be counted, and the limitations of the study arising from the selection criteria.

**Insert: Page 7, Line 110–Page8, Line 118**
Local governments where public health centers are located consist of prefectures that unite cities, towns, and villages, and cities with large populations, such as ordinance-designated cities with populations of 500,000 or more and core cities with populations of 200,000 or more. Including other smaller cities, towns and villages, there are 1,718 local governments in Japan. However, only local government with public health centers were included in this survey because large local governments, which have more discretion over personnel costs, are more sensitive to increasing or decreasing the number of PHNs, and public health centers are also likely to be significantly affected by COVID-19 pandemic.

**Insert: Page 8, Line 122–124**
However, since the objective of this study was to determine the number of staff engaged in public health nursing services, we requested that PHNs and RNs working in hospitals and clinics operated by local governments be excluded from the count.

**Insert: Page 25, Line 364–368**
Thirdly, the survey for local governments conducted in this study was conducted for prefectures and large cities and did not take into account the number of PHNs in smaller local governments. However, smaller local governments were excluded from the study because some of them have no choice for increase or decrease in the number of PHNs, with only 1 or 2 PHNs assigned to them in the first place, and they may be affected by other factors, such as the shortage of human resources in rural areas.

**#2_4. In addition to the above, describe the characteristics of the personnel who were responding to the questionnaire at the site.
Response:** Thank you for your important suggestion. We added the sentences to describe the characteristics of personnel who were responding the questionnaire.

**Insert: Page 7, Line 102–106**
We identified from the target local governments' websites the department in charge of human resource management, such as recruitment, leave of absence, retirement, and staffing, and asked the department preparing the responses for questionnaire. In the Japanese government system, the responses were generally double-checked by the staff and manager in the department and the answer was given in the name of the head of the department.

**#2_5. There is limited information provided on the contents of the questionnaire which was used in the study. The questionnaire should be included as supplementary file
Response:** Yes, I have attached the questionnaire as Supplementary file 4 (S4). However, due to the large volume of the entire survey instrument, and in order to avoid leakage of research ideas and to be considerate of items that require permission for publication, we have excerpted only those items that are relevant to the analysis of this study.

| **Original: Page 7, Line 101 – 104** the survey items included the number of full-time PHNs hired, number of new graduates among the full-time PHNs hired, number of part-time PHNs hired, and number of full-time PHNs who quit or retired. | **Revised: Page 8, Line 119 – 122** the survey items included the number of full-time PHNs hired, number of new graduates among the full-time PHNs hired, number of part-time PHNs hired, and number of full-time PHNs who quit or retired (S4). |
| --- | --- |
| **Insert: Page 33, Line 494 – 495** S4. Survey on the Employment Status of Public Health Nurses (PHNs) during the Corona Disaster Survey Questionnaire | |

**#2_6. In the results, number of PHN is presented in decimals. For example line 215 (103.2) and in table 1. May you explain this as it is confusing to the reader
Response:**　 I appreciate your honest comments. We had received similar comments in Reviewer comment #1_5, so we revised the sentences to clearly convey our meaning.

| **Original: Page 14, Line 214 – 218** The response rate of the survey was 54.0% (81/150). For each variable, we compared the data for 2019 and 2020, that is, before and after the COVID-19 pandemic, respectively. The number of full-time PHNs was 103.2 ± 75.9 in 2019 and 104.5 ± 76.4 in 2020. The number of part-time PHNs was 11.7 ± 14.9 in 2019 and 22.4 ± 53.6 in 2020. The number of PHNs who quit or retired was 5.0 ± 4.3 in 2019 and 4.9 ± 4.5 in 2020. The number of PHN recruitment examinations was 1.3 ± 1.0 in 2019 and 1.6 ± 1.1 in 2020 (Table 1). | **Revised: Page 15, Line 230 – 236** The response rate of the survey was 54.0% (81/150). For each variable, we compared the data for 2019 and 2020, that is, before and after the COVID-19 pandemic, respectively. The mean number of full-time PHNs for local governments was 103.2 (Standard Deviation: SD=75.9) in 2019 and 104.5 (SD=76.4) in 2020. The mean number of part-time PHNs for local government was 11.7 (SD=14.9) in 2019 and 22.4 (SD=53.6) in 2020. The mean number of PHNs who quit or retired for local government was 5.0 (SD=4.3) in 2019 and 4.9 (SD=4.5) in 2020. The number of PHN recruitment examinations for local government was 1.3 (SD=1.0) in 2019 and 1.6 (SD=1.1) in 2020 (Table 1). |
| --- | --- |

**#2_7. Are the numbers presented in table 1 for the whole country? Provide further details to help readers interpret this table, preferably in as table legend
Response:** Thank you for your useful question. We modified Table 1, including the title, spelling out the abbreviations as much as possible and writing explanations carefully (Table 1).

**#2_8. In line 255, include the n in blackets after the %
Response:** Yes, we have added the number of public health nurses you indicated in brackets after the %.

| **Original: Page 17, Line 255–257** In 2020, approximately 1.2% of people from the human resource pool changed jobs to PHNs, and the estimated number of people changing jobs to PHNs at the same rate would be 1,557 in 2025, 1,151 in 2030, and 1,180 in 2035. | **Revised: Page 19, Line 274–276** In 2020, approximately 1.2% (n=1,948) of people from the human resource pool changed jobs to PHNs, and the estimated number of people changing jobs to PHNs at the same rate would be 1,557 in 2025, 1,151 in 2030, and 1,180 in 2035. |
| --- | --- |

**#2_9. Line 269, this sentence is misplaced
Response:** We apologize for the misplacement of the sentence you pointed out. The exact same sentence is on Page 19, Line 274 and we removed the sentence.

**Removed: Page 18, Line 269**
Moreover, the number of PHNs newly hired in 2020 was 2,185.

**#2_10. In figure 1, I do not understand what NOT PHN and potential PHN means. Is NOT PHN everyone else who is not a PHN? Provide more information in a legend
There are more than three graphs in figure 1 and 2. Consider labelling them as 1A, 1B, 1C….
Response:** We completely agree with your suggestions.　 In the footnote of Fig. 1, we added explanations for "NOT PHNs" and "Potential PHNs". In addition, the panels in Figs. 1 and 2 are now labeled with numbers and subscripts A, B, and C (Fig1, Fig2).

**Insert: Footnotes for Fig 1**
Note. “NOT PHNs” refers to the total number of persons who have a national PHN licenses and are not currently employed as a PHNs. In addition, “Potential PHNs” refers to the number of NOT PHNs who are under the upper average age limit for employment requirements set by the local government.

**#2_11. In addition to the above suggestion, think about it if it is necessary to have a separate graphs for estimates at 2025, 2030 and 2035. I am of the view that one graph can tell the whole story in a sequence
Response:** We agree with you very much but could not combine the three panels of Fig1 and Fig 2 into one because it would make the figures very complex and would make it difficult to understand the estimation process of supply and demand. However, we added Fig. 4 because we believe that the figure on the gap between demand and supply of PHNs will help readers better understand the whole story of this study. Accordingly, the results of Fig. 4 have been added to the abstract and main text.

| Original: Abstract At the current job change rate, the supply of PHNs is expected to be 2,285 in 2025, 1,879 in 2030, and 1,908 in 2035. The demand for PHNs is estimated to be 2,335–2,779 in 2025, 2,314–2,886 in 2030, and 2,238–2,680 in 2035. | Revised: Abstract At the current job change rate to PHNs, the gaps between future supply and demand of PHNs were expected to be -494 – -50 in 2025, -1,007 – -435 in 2030, and -772 – -330 in 2035. If the job change rate would be 1.5 times, the gaps were estimated to be 285 – 729 in 2025, -31 – 141 in 2030, and -182 – 260 in 2035. If the job change rate would be 2.0 times, the supply was sufficient for all estimated years. |
| --- | --- |
| Insert: Page 20, Line 292–298  Calculating the gap between supply and demand for future PHNs, the supply was insufficient for demand in all estimated years 2025, 2030, and 2035 when the current rate of job change to PHNs was used. If the job change rate to PHNs would be increased by 1.5 times, the supply was sufficient in 2025, but in 2030 and 2035, there was a shortage of up to 431 positions. Furthermore, if the job change rate would be increased to 2 times, the supply was sufficient for all estimated years (Fig 4).  Fig 4. Gaps of future supply and demand of public health nurses. | |

**#2_12. Provide title for figure 2 and figure 3**

**Response:** We apologize for the omission of the titles in the figure files. We wrote the title in the figure insertion area of the main text, but we also added it to the figure file as well.

**List of small modifications other than the above comments**

・In accordance with the PLOS ONE submission guidelines, we have revised the figure labels "Figure1" to "Figure 4" were corrected to "Fig1" to "Fig4".

・Since "supply and demand" and "demand and supply" were mixed in the text, we unified the entire text to "supply and demand" to match the title.

・As with the response to the reviewer comment #2_8, the number of samples (n) was added where only percentages are shown.

| **Original: Page 16, Line 247–248** The percentage of total PHNs aged 60 and older almost doubled, from 3.6% in 1996 to 8.1% in 2020. | **Revised: Page 19, Line 274–276** The percentage of total PHNs aged 60 and older almost doubled, from 3.6% (n = 1,142) in 1996 to 8.1% (n = 4,518) in 2020. |
| --- | --- |

・As with responses to the reviewer comments #1_5 and #2_6, the manuscript has been revised to clearly indicate that the numerical values presented in the respective sentences represent means and standard deviations.

| **Original: Page 15, Line 231 – Page 16, Line 237** The average number of recruitment information posted on “Komuin” from 2013 to 2020 was 1,009 ± 125. Japan has 47 prefectures and 1,718 municipalities for 1,765 local governments, and the coverage rate was roughly 57.2% (1,009/1,765). PHN recruitment information by local governments showed a similar bimodal pattern every year (S1). The average age limit for the employment of PHNs calculated from the recruitment information was 34.1 ± 5.7 years in 2013, 36.2 ± 6.7 years in 2017, and 37.5 ± 7.2 years in 2021, reflecting an upward trend. | **Revised: Page 17, Line 241 – 247** The mean number of recruitment information posted on “Komuin” from 2013 to 2020 was 1,009 (SD=125.) Japan has 47 prefectures and 1,718 municipalities for 1,765 local governments, and the coverage rate was roughly 57.2% (1,009/1,765). PHN recruitment information by local governments showed a similar bimodal pattern every year (S1). The average age limit for the employment of PHNs calculated from the recruitment information was 34.1(SD=5.7) years in 2013, 36.2 (SD=6.7) years in 2017, and 37.5 (SD=7.2) years in 2021, reflecting an upward trend. |
| --- | --- |
