# Supplementary material for: Estimation of supply and demand for public health nurses in Japan: A stock-flow approach
Source: PLoS One. 2025 Feb 3;20(2):e0313110. doi: 10.1371/journal.pone.0313110 (PMC11790149; doi:10.1371/journal.pone.0313110)
Supplement: Attachment — Submitted filename: List_of_Changes.docx [file pone.0313110.s006.docx]

Dear Editors and Reviewers,

Thank you for allowing us the opportunity to review our manuscript. Also, we are glad to have constructive and educational comments from you. We believe that our manuscript has been considerably improved thanks to your comments.

Below are our point-by-point responses to your comments and the manuscript with track change is uploaded as a supplementary file. Also, we found several other improvements in addition to the points suggested in the peer review, which we have also corrected. The corrections are summarized at the end of the document. Your comments have made the paper much better and more readable for the readers. We would appreciate it if you could check the revisions.

**Response to Reviewer #4**

**#1. Line 89: The questionnaire(s) links/attachment(s) is/are to be cited.
#2.** **Line 123, 130, 131-134: The weblink is to be provided.
#7. Line 161: The website link is to be inserted.
#9. Line 209: The link is to be provided.
#15. Line 272: The figure is to be cited**
**Response:** The reviewer comments (#1, 2, 7, 9 and 15) suggested adding links. While the submission guidelines for PLOS ONE do not explicitly mention this (URL), they instruct authors to use the Vancouver citation style. In this style, it is generally understood that URLs should be included in the reference list at the end of the manuscript. Accordingly, we have added citations with numbers where applicable but have not embedded URLs directly into the main text. If my understanding is incorrect, I would be happy to make the necessary adjustments, so please feel free to provide further instructions.

| **Original: Page 6, Line 89–90** This was a simulation study based on a questionnaire survey of Japanese local governments, secondary data from government statistics, and log data from a website. | **Revised: Page 6, Line 89–90** This was a simulation study based on a questionnaire survey of Japanese local governments, secondary data from government statistics, and log data from a website [16–21]. |
| --- | --- |
| **Original: Page 10, Line 159–Page 11, Line 161** The age limit was set at 37 years, which is the average upper age limit for the recruitment examinations for PHNs calculated from the log data from the recruitment information website. | **Revised: Page 11, Line 160–161** The age limit was set at 37 years, which is the average upper age limit for PHN　recruitment examinations, calculated from the log data in recruitment information [16]. |
| **Original: Page 17, Line 270–274** The future number of PHNs needed, which is estimated based on the regression equation applied to each bivariate, is 58,567 in 2025, 60,251 in 2030, and 62,260 in 2035 based on the number of births; 13,397 in 2025, 16,904 in 2030, and 19,380 in 2035 based on the number of persons requiring certified long-term care; and 56,347 in 2025, 56,990 in 2030, and 57,257 in 2035 based on the estimated number of patients with cancer (S3, Fig 2). | **Revised: Page 11, Line 273–277** The future number of PHNs needed, which is estimated based on the regression equation applied to each bivariate, is 58,567 in 2025, 60,251 in 2030, and 62,260 in 2035 based on the number of births [20]; 13,397 in 2025, 16,904 in 2030, and 19,380 in 2035 based on the number of persons requiring certified long-term care [21]; and 56,347 in 2025, 56,990 in 2030, and 57,257 in 2035 based on the estimated number of patients with cancer [22] (S3_Fig, Fig 2). |

**#3. Line 180-186: The equations could be fine-tuned to the following:**

- **Demand Estimate at Ti=The number of additional public health nurses needed at Ti + The number of new public health nurses hired by local governments in 2020.**
- **The number of additional public health nurses needed at Ti =The projected number of public health nurses needed at Ti − The number of public health nurses employed by local governments in 2020.**
- **The projected number of public health nurses needed at Ti = [Regression coefficient × Demographic parameters at Ti] +Intercept.**

**Response:** Based on the fine-tuned equations you suggested, I have revised the formulas for demand calculations.

| **Original: Page 12, Line 180–186** Demand Estimates Ti＝The number of increased PHNs demand at point Ti　+ The number of new PHNs hired by local governments in 2020  The number of increased PHNs demand at point Ti = The number of PHNs that would be needed at point Ti − The number of PHNs working in local government in 2020  The number of PHNs that would be needed at point Ti **=** Regression coefficient [B] × the demographic parameters Ti + [Intercept] | **Revised: Page 12, Line 183–189** Demand Estimates at Ti＝The number of additional public health nurses needed at Ti+ The number of new public health nurses hired by local governments in 2020  The number of additional public health nurses needed at Ti = The projected number of public health nurses needed at Ti − The number of public health nurses employed by local government in 2020  The projected number of public health nurses needed at Ti **=** [Regression coefficient × Demographic parameters at Ti] + Intercept |
| --- | --- |

**#4. Line 139 -147: The equations could be fine-tuned to the following:**

- **Supply Estimate at Ti=The number of newly graduated public health nurses at Ti + The number of people transitioning to public health nursing roles at Ti.**
- **The number of people transitioning to public health nursing roles at Ti = The potential public health nurse resource pool at Ti × Percentage of job changers entering public health nursing.**
- **The human resource pool of potential public health nurses at Ti = The number of nurses within the employable age range at Ti−The number of nurses already working as public health nurses.**

**Response:** Based on the fine-tuned equations you suggested, I have revised the formulas for supply calculations.

| **Original: Page 9, Line 139–Page 10, Line 147** Supply Estimates Ti ＝The number of newly graduated PHNs at point Ti + The number of people changing jobs to PHNs at point Ti    The number of people changing jobs to PHNs at point Ti*=*The human resource pool of potential PHNs at point Ti  × The percentage of job changes to PHNs  The human resource pool of potential PHNs  =The number of PHNs within the age limit for employment at point Ti − The number of nurses already working as PHNs | **Revised: Page 9, Line 139–Page 10, Line 147** Supply Estimates at Ti ＝The number of newly graduated public health nurses at Ti + The number of people transitioning to public health nurses at Ti  The number of people transitioning to public health nurses at Ti =The potential public health nurse resource pool at Ti × Percentage of job changers entering public health nursing  The potential public health nurse resource pool at Ti =The number of nurses within the employable age range at Ti − The number of nurses already working as public health nurses |
| --- | --- |

**#5. The numbering of Parameters 1-4 and Parameters 1-3 requires revision. If different, different number is to be given.
Response:** Since the parameters related to supply (Parameter 1-4) and those related to demand (Parameter 1-3) were mixed, I have renamed them as 'Supply-related Parameter 1-4' and 'Demand-related Parameter 1-3.'

**#6. Line 159-161: The statement could be revised as ‘****The age limit was set at 37 years, which is the average upper age limit for public health nurse recruitment examinations, calculated from the log data in recruitment information.’
Response:** Thank you for your thoughtful suggestions on improving the English expressions. I agree with your suggestions and have revised the main text accordingly.

| **Original: Page 10, Line 159–Page 11, Line 161** The age limit was set at 37 years, which is the average upper age limit for the recruitment examinations for PHNs calculated from the log data from the recruitment information website. | **Revised: Page 11, Line 160–161** The age limit was set at 37 years, which is the average upper age limit for PHN　recruitment examinations, calculated from the log data in recruitment information [16]. |
| --- | --- |

**#8. Line 167-168: The statement could be improved as ‘The number of employed public health nurses within a five-year age group in 2020 was obtained from government statistics to estimate the number of nurses already working as public health nurses."
Response:** Thank you for your thoughtful suggestions on improving the English expressions. I agree with your suggestions and have revised the main text accordingly.

| **Original: Page 11, Line 167–168** The number of employed PHNs within a five-year age group in 2020 was taken from government statistics to estimate the number of nurses already working as PHNs [19]. | **Revised: Page 11, Line 169–161** The number of employed PHNs within a five-year age group in 2020 was obtained from government statistics to estimate the number of nurses already working as PHNs [19]. |
| --- | --- |

**#10. Line 213: ‘Them’ could be replaced with ‘total demands for 5 years’ where total demands for 5 years divided by 5
Response:** Thank you very much for your suggestion. I determined that avoiding pronouns would improve readability, so I have revised the text as per your recommendation.

| **Original: Page 14, Line 213–214** Since only five-year estimates could be calculated, we divided them by five and treated the average as the estimate for each year. | **Revised: Page 14, Line 216–217** Since only five-year estimates could be calculated, we divided the total demand for five years by five and treated the average as the estimate for each year. |
| --- | --- |

**#11. Table 1: IQR could be used in place of ‘minimum – maximum’.
Response:** Thank you for your insightful suggestion. I understand that it is common practice to report the IQR along with the median. However, in order to highlight the reality in certain local governments where the working environment was very challenging, I decided that including the maximum and minimum values would provide a clearer picture. Therefore, I have revised the table to include both the IQR and the maximum-minimum values.

**#12. Line 241: Dot is to be placed after close bracket for (SD=125.) Japan
Response:** I apologize for the typo regarding the placement of the period. I have reviewed and corrected the indicated sentence as well as the entire text.

**#13. Line 261: ?37 (unclear)
Response:** Thank you for your important feedback. Since the meaning of 'the number of PHNs under 37' was unclear, I have made the following revision.

| **Original: Page 18, Line 259–262** For the number of people changing their job to PHNs, first, the number of PHNs under 37 is estimated to be 157,704 in 2025, 125,733 in 2030, and 116,752 in 2035. | **Revised: Page 18, Line 263–267** For the number of people changing their job to PHNs, first, the number of PHNs within the employable age range is estimated to be 157,704 in 2025, 125,733 in 2030, and 116,752 in 2035. |
| --- | --- |

**#14. S1, S2, S3, S4 is to be named as S1_Fig, S2_Fig, S3_Fig, S4 _Fig
Response:** To ensure consistency with the Supporting Information Captions, I have updated the references in the main text to S1_Fig, S2_Fig, S3_Fig, and S4_Fig.

**#16. Figure 3: Data in 2025, 2030, and 2035 could be displayed in the figure.
Response:** Thank you for your feedback. I have added numerical values to the Figure 3.

**#Other revisions:**

- The link for Reference 2 was broken, so it has been updated with a new one.
- An error was identified in the in-text citation numbering, where [29] was missing. This has been corrected.
- As a result of inserting the missing citation, the numbering for subsequent citations throughout the manuscript has been adjusted accordingly.
